# A Path to Discovery: The Career of Maclyn McCarty

**DOI:** 10.1371/journal.pbio.0030341

**Published:** 2005-10-11

**Authors:** Joshua Lederberg, Emil C Gotschlich

## Abstract

Maclyn McCarty (1911-2005) was best known for his part in the pioneering discovery that genes are made of DNA.

Maclyn McCarty, who devoted his life as a physician-scientist to studying infectious disease organisms, was best known for his part in the monumental discovery that DNA, rather than protein, constituted the chemical nature of a gene. Uncovering the molecular secret of the gene in question—that for the capsular polysaccharide of pneumococcal bacteria—led the way to studying heredity not only through genetics but also through chemistry, and initiated the dawn of the age of molecular biology. McCarty was the youngest and longest surviving member of the research team responsible for this feat, which also included Oswald T. Avery and Colin MacLeod; he died on January 2, 2005, from congestive heart failure.

McCarty was born in 1911 in South Bend, Indiana, the second of four sons of a branch manager for the Studebaker Corporation while it was still a firm for horse-drawn carriages. In his teens, McCarty set himself the goal of becoming a physician-scientist, and he pursued a successful strategy to prepare for admission to, and early success in, Johns Hopkins University Medical School. As an undergraduate at Stanford University, he presciently began his studies in the nascent field of biochemistry, working with James Murray Luck on protein turnover in the liver. In 1937, he began his clinical training in pediatrics at the Harriet Lane Service at Johns Hopkins University. There McCarty developed a special interest in infectious diseases—in particular, antibacterial sulfonamide drug treatments that were just entering medicine—which he subsequently pursued by moving to New York University to work with William Tillett. A National Research Council Fellowship in the medical sciences and an opening in Oswald T. Avery's laboratory spurred his move to Rockefeller University in 1941.

At that time, research in the Avery laboratory was focused on the pneumococcal transformation, the heritable alteration of a pneumococcal strain from a nonvirulent rough form to a virulent smooth encapsulated form. McCarty's arrival at the Rockefeller Institute in September 1941 marked 13 years since this discovery, also known as the Griffith phenomenon. Prior to this discovery, the 1920s had been marked by a medley of disparate observations on Streptococcus pneumoniae that seemed to involve an exchange of receptors among diverse bacteria either grown together in liquid media or exposed to various kinds of extracts and supernatants. With rare exception, the early researchers in this area were utterly confused about the distinction between genotype and phenotype. No single experiment was carried forward to confirmation by other observers, so the entire field of “para agglutination” was in some disrepute. [Fig pbio-0030341-g001]

**Figure pbio-0030341-g001:**
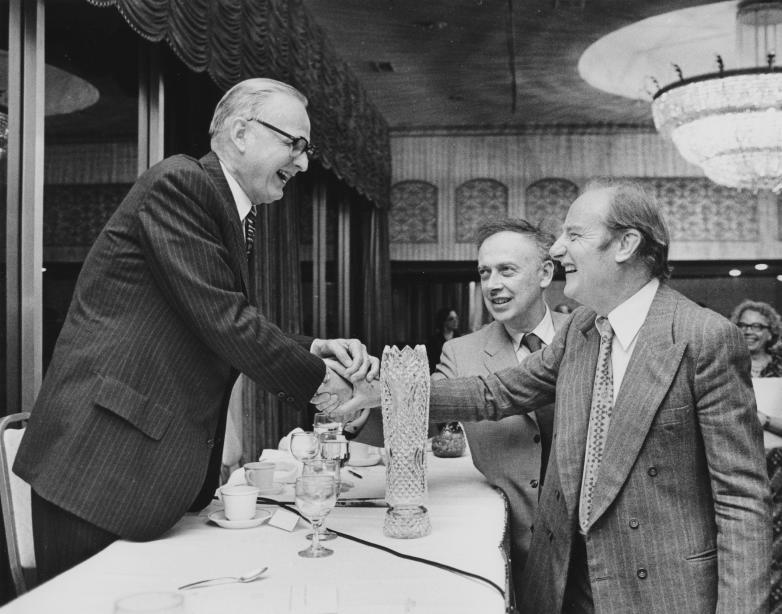
Maclyn McCarty (June 9, 1911, to January 2, 2005) with Francis Crick and James D. Watson (Photo: Marjorie McCarty)

However, in 1928, Fred Griffith, a leader in public-health research in Britain, demonstrated that the conversion of one strain to another could happen in vivo in mice. Shortly after the publication of his results, they were confirmed in several quarters, including Avery's lab. The analysis relied on serotyping: it was known that phenotypic differentiation of pneumoccocal groups could be diagnosed by their reactions with specific antisera, already recognized to reflect chemically distinct capsular polysaccharides. Griffith had neither the resources nor the inclination to purify and identify the responsible agent in pneumococcal extracts that induced the changes of serotype. But the phenomenon of transformation was at least vaguely understood to comprise an alteration of what we would now call genetic factors.

Though interrupted, sometimes for years at a time, these studies were from 1928 onwards the centerpiece of Avery's lab agenda. Around 1940, they were activated by Colin MacLeod's efforts to purify the chemical agent responsible for changes of serotype—whether protein, nucleic acid, or some other class of molecule—and demonstrate that it was necessary and sufficient to cause the Griffith phenomenon. Studies on pneumococcal transformation were grossly burdened by a wide variety of variables, which needed to be controlled to allow quantitative estimation of transforming activity in extracts undergoing various stages of purification. MacLeod, over a number of years of research, had resolved several thorny technical issues to render the experimental system somewhat more reliable as an assay for biological activity. By the time McCarty arrived at the Rockefeller University, Avery's team had just about decided that the active reagent was not a protein. But what was it then? Could it be a soluble saccharide, RNA, or, least likely, DNA? The progress of this research over the next three years is beautifully described in McCarty's memoir *The Transforming Principle*, written in the early 1980s [1].

As purification progressed, exposure of extracts to crystalline RNase and to proteinase preparations helped Avery's team determine that the biological activity of extracts was not dependent on RNA or protein. Crystalline DNase was not available until 1948, but biological activity was rapidly reduced by tissue extracts rich in DNase. McCarty's arrival at Rockefeller University was also marked by another milestone, namely, the development of a diphenylamine reagent assay to positively correlate DNA with biological activity. It gradually became evident that the active material in purified extracts had astonishingly high potency in micrograms of DNA that could consummate the pneumococcal transformation in vitro.

McCarty, MacLeod, and Avery wrestled with the standard of proof required to claim that they had accomplished pneumococcal transformation with highly purified DNA from extracts. After much self-inquiry, in 1944, they published in the *Journal of Experimental Medicine* that the active material was, indeed, DNA [2], bereft of protein or any other known polymer [3,4].

The vicissitudes of the acceptance of the concept that “genes are DNA” deserve the scholarly praise they have received [5,6]. The claim was, indeed, subject to a formidable, but predictable, round of organized skepticism. Some would say, even worse, that it was simply ignored, but that is manifestly untrue, at least in the case of the New York research institutions. The scientific community does not accept major scientific claims with ease, and in this case, there were challenges associated with research on S. pneumoniae, which made it especially difficult to attract other investigators to pursue this research. To begin with, few people had the necessary expertise with this pathogen from a biological perspective—it was dangerous to work with, and at the same time, it was finicky to grow. In order to assay its virulence, one needed to use mice as a selective filter. Most critically lacking as corroboration was the examination of other phenotypic markers, besides the capsular polysaccharide, to determine the extent that the findings on the gene for one pneumococcal antigen would apply to other metabolic markers of S. pneumoniae.

However, by 1953, influenced by the enormous impact of Watson and Crick's bihelical structure of DNA, the majority of researchers had fully accepted the 1944 paper. In fact, one might say, formal proof that DNA encoded genetic material was approximated only much later by the laboratory synthesis of oligonucleotides, and by the demonstration of genetic material's biological activity, for example, genes for tRNA or small DNA viruses. Long before this formal proof, most commentators had accepted the untrammeled heuristic value of the proposition that, indeed, genes were made of DNA.

Meanwhile, a physician-scientist through and through, McCarty turned his attention to diseases promoted by streptococci. So it happened that on the retirement of Homer Swift in 1946, McCarty was asked to head the laboratory established in 1922 to work on streptococci and rheumatic fever. This was the scientific home of Rebecca Lancefield, who developed the still powerful serological classification of streptococci. From innumerable clinical observations, combined with Lancefield's classification, it was clear that acute rheumatic fever, a severe sterile inflammatory condition affecting particularly the joints and the heart, was a complication of group A streptococcal pharyngitis, following the infection by several weeks. The causal chain of events still eludes us. McCarty attacked this problem by studying both the biology of group A streptococci and patients with acute rheumatic fever admitted to the Rockefeller Hospital.

Together with his students and collaborators, over the next 20 years, McCarty's work changed the understanding of the organism from a gram-positive streptococcus with a particular serological characteristic to one of the best characterized bacterial species. Work on bacterial cell-wall anatomy and chemistry was just beginning. His work led to the isolation of the streptococcal cell wall as a structural entity suitable for anatomic inspection by electronmicroscopy. Chemical dissection led to characterization of the group A–specific polysaccharide and the peptidoglycan, and the identification of its serological specificity in the terminal hexosamine. In order to prove this specificity, he first had to identify and purify a specific enzyme that cleaved hexosamine (a hexosaminidase) from a soil organism. Treating the polysaccharide with this enzyme abrogated its serological reactivity. McCarty further demonstrated the precise configuration of the hexosamine linkage by synthesizing both α- and β-N-acetyl-glucosamine ovalbumin and showing that only the second reacted with group A antisera. A similar analytical strategy indicated that the polysaccharide of group C streptococci differed by having a terminal β-N-acetyl galactosamine as the serological determinant.

In parallel, McCarty studied patients with rheumatic fever admitted to the Rockefeller Hospital as well as valuable specimen collections from military outbreaks of the disease during World War II. He and his collaborators found that antibody responses to several streptococcal antigens were significantly higher in the group of individuals that developed acute rheumatic fever than in individuals with uncomplicated infection. However, the response to unrelated antigens, for instance, diphtheria toxoid, was not enhanced. He found that group A streptococci secreted unusually high amounts of DNase, and established a test for the detection of antibodies produced in response to this antigen. This led to the discovery that streptococci were able to produce multiple isozymes of DNase. He purified human C-reactive protein through crystallization, produced a highly specific antiserum, and, using this much simpler and more sensitive test, found that C-reactive protein levels responded more rapidly and reliably than other inflammatory markers and could serve as the most accurate indicator of rheumatic inflammatory activity. Measuring C-reactive protein levels to detect inflammation is routine now in medical practice.

In his later years, McCarty increasingly served as a statesman of the biomedical sciences. He served for 14 years as the physician-in-chief of the Rockefeller University Hospital, and as a trusted adviser and the vice president of the Rockefeller University. Outside the university, his leadership was sought by the New York City Health Research Council, the Helen Hay Whitney Foundation, the Institute of Medicine (as a charter member), and numerous university visiting boards. For more than 40 years, as editor, he placed his stamp of excellence and integrity on the *Journal of Experimental Medicine*.

McCarty's scientific interests and energy had a counterpart in his rich personal life. Along with his wife, Marjorie, McCarty had a wide circle of very close friends, both in the United States and abroad, who cherished his personal warmth, his low key, spare, and pragmatic character, his wit, and his wide-ranging intellect. He loved English literature, theater, and symphonies. He loved to wander the streets and the museums of the great cities of the world, particularly, Paris, New York, and London, and frequently visited overseas following his retirement. Moreover, he remained close to his family; the four brothers, living in different parts of the country, never failed to meet for annual reunions. McCarty lived a full life, and he continues to inspire all who knew him.
